# Andrographolide: Mechanisms and Therapeutic Potential in Alzheimer’s and Parkinson’s Disease

**DOI:** 10.3390/molecules31183139

**Published:** 2026-09-08

**Authors:** Angélica Ríos-Gallardo, Daniela Herrera-Ramirez, Sussy Bastias-Candia, Nibaldo C. Inestrosa

**Affiliations:** 1 Centro de Excelencia en Biomedicina de Magallanes (CEBIMA), Universidad de Magallanes, Punta Arenas 6200000, Chile; angelica.rios@umag.cl (A.R.-G.); daniela.herrera@umag.cl (D.H.-R.); 2 Escuela de Medicina, Universidad de Magallanes, Punta Arenas 6200000, Chile; 3 Facultad de Ciencias Biológicas, Pontificia Universidad Católica de Chile, Av. Bernardo O’Higgins 340, Santiago 8331150, Chile

**Keywords:** neurodegenerative diseases, natural compounds, andrographolide, proteostasis, Wnt/β-Catenin signaling, GSK-3β, cognition, memory

## Abstract

Neurodegenerative diseases such as Alzheimer’s disease (AD) and Parkinson’s disease (PD) are characterized by the progressive loss of specific neuronal cell populations and are associated with protein aggregates. Current therapeutic approaches are still limited due to the complexity and heterogeneity of these diseases, which points toward an urgent need to discover and develop new therapeutic agents. Natural compounds are a promising source of novel bioactive agents targeting multiple mechanisms of action implicated in neurodegeneration. Andrographolide (ANDRO) is a natural compound extracted from *Andrographis paniculata*, a traditional Chinese herb known for its anti-inflammatory and antioxidant properties, which has emerged as a potential neuroprotective agent due to its ability to cross the blood–brain barrier (BBB). ANDRO can exert neuroprotective effects by modulating numerous transcription factors and signaling pathways across different cell types in the central nervous system (CNS). It has been described that ANDRO reverses cognitive and/or motor impairments in AD and PD study models. However, the cellular and molecular mechanisms behind these protective effects are still being elucidated. In this review, we analyze the most recent findings on ANDRO, a neuroprotective agent with multiple biological targets that could reduce the progression of the most prevalent neurodegenerative diseases, AD and PD.

## 1. Introduction

**Figure 1:**
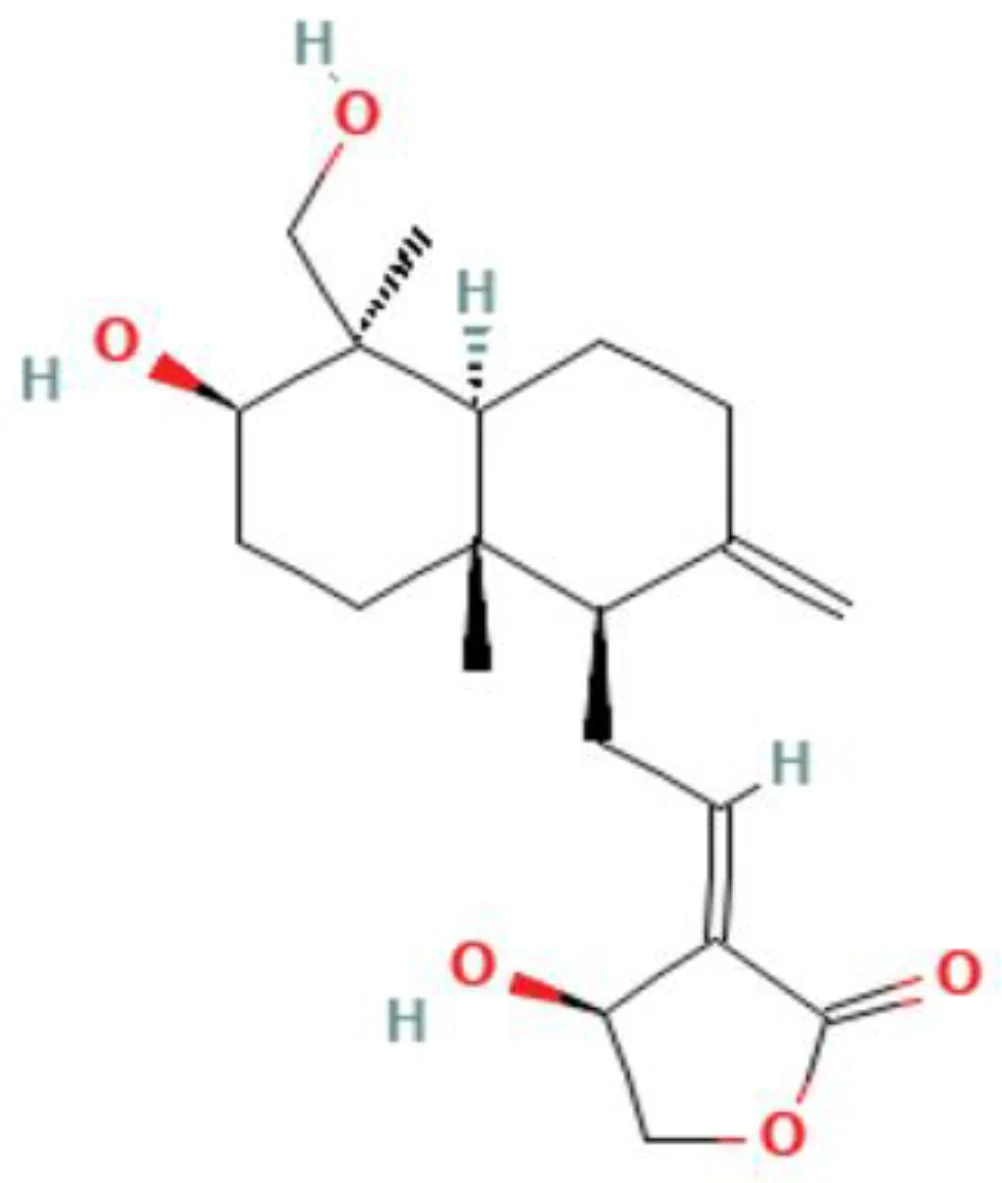
ANDRO molecular structure. The figure shows chemical structure of (3E,4S)-3-[2-[(1R,4aS,5R,6R,8aS)-6-hydroxy-5-(hydroxymethyl)-5,8a-dimethyl-2-methylidene-3,4,4a,6,7,8-hexahydro-1H-naphthalen-1-yl]ethylidene]-4-hydroxyoxolan-2-one known as ANDRO.

*Andrographis paniculata* (Burm.f.) Wall. ex Nees (*A. paniculata*) is a traditional plant that has been used for centuries in Asian countries as a natural treatment for inflammatory processes like rheumatoid arthritis, fever, and upper respiratory tract infections [1]. The aerial part of the plant, which is used medicinally, has many bioactive compounds, such as lactones, diterpenoids, and flavonoids [2,3]. The primary bioactive phytochemical of *A. paniculata* is the diterpenoid lactone Andrographolide (ANDRO) [3,4], with the molecular formula C_20_H_30_O_5_ (PubChem: 5318517) (Figure 1). It has been reported to have a wide range of therapeutic actions, including hypoglycemic [5,6], hepatoprotective [7], immunomodulator [8], antimicrobial and antiparasitic properties [9], as well as antioxidant and anti-inflammatory activities [10]. Studies in rodents indicate that ANDRO effectively crosses the blood–brain barrier (BBB) because it is a highly lipid-soluble, low-molecular-weight nonpolar compound [11,12,13]. These characteristics make ANDRO a candidate for the treatment of neurodegenerative diseases. Therefore, its pharmacological effects at the CNS level have been studied in recent years [14]. Regarding pharmacokinetic studies, in Sprague–Dawley rats, oral administration of 50 mg/kg ANDRO produces a maximum plasma concentration of 1 mM at 30 min of administration, and the bioavailability is 1.19% [15]. In human plasma samples, 200 mg of ANDRO generates a maximum plasma concentration of 58.32 ng/mL at 1.6 h post-administration, and the elimination half-life is 10.50 h [16]. However, this low bioavailability is overcome using ANDRO nanocrystals and nanoparticles and other nano-formulations [13,17].

Numerous studies have demonstrated that ANDRO exhibits neuroprotective effects by modulating several proteins and signaling pathways. In vitro studies in neuronal and microglial cultures showed that ANDRO promoted anti-inflammatory and antioxidant activity via activation of Nuclear factor erythroid 2-related factor 2 (Nrf2) mediated signaling pathways and inhibition of the JNK, MAPK, and NF-κB p65 pathways [18,19,20]. In addition, ANDRO reduced the expression of proinflammatory cytokines (TNF-α, IL-1β, and IL-6) and increased the activity of antioxidant enzymes superoxide dismutase (SOD), catalase (CAT), and glutathione (GSH) in microglial cell cultures and animal models [19,21,22]. Furthermore, ANDRO is a potent modulator of the Canonical Wnt/β-catenin signaling pathway in hippocampal neurons, which plays a role in adult neurogenesis and regulates synaptic structure and function [23,24]. Interestingly, ANDRO has been reported to attenuate BBB disruption [25]. 

Taken together, these studies suggest that ANDRO could modulate several signaling pathways and exert protective effects across different cell types, such as microglial and hippocampal cells, in the CNS. Due to these properties, ANDRO is being studied as a treatment for neurodegeneration. In this review, we summarize the neuroprotective molecular mechanisms of ANDRO, focusing on studies conducted in cellular and animal models of the most important neurodegenerative diseases: AD and PD.

## 2. Neuroprotective Effects of ANDRO in Alzheimer’s Disease

### 2.1. Pathophysiology of Alzheimer’s Disease

**Figure 2:**
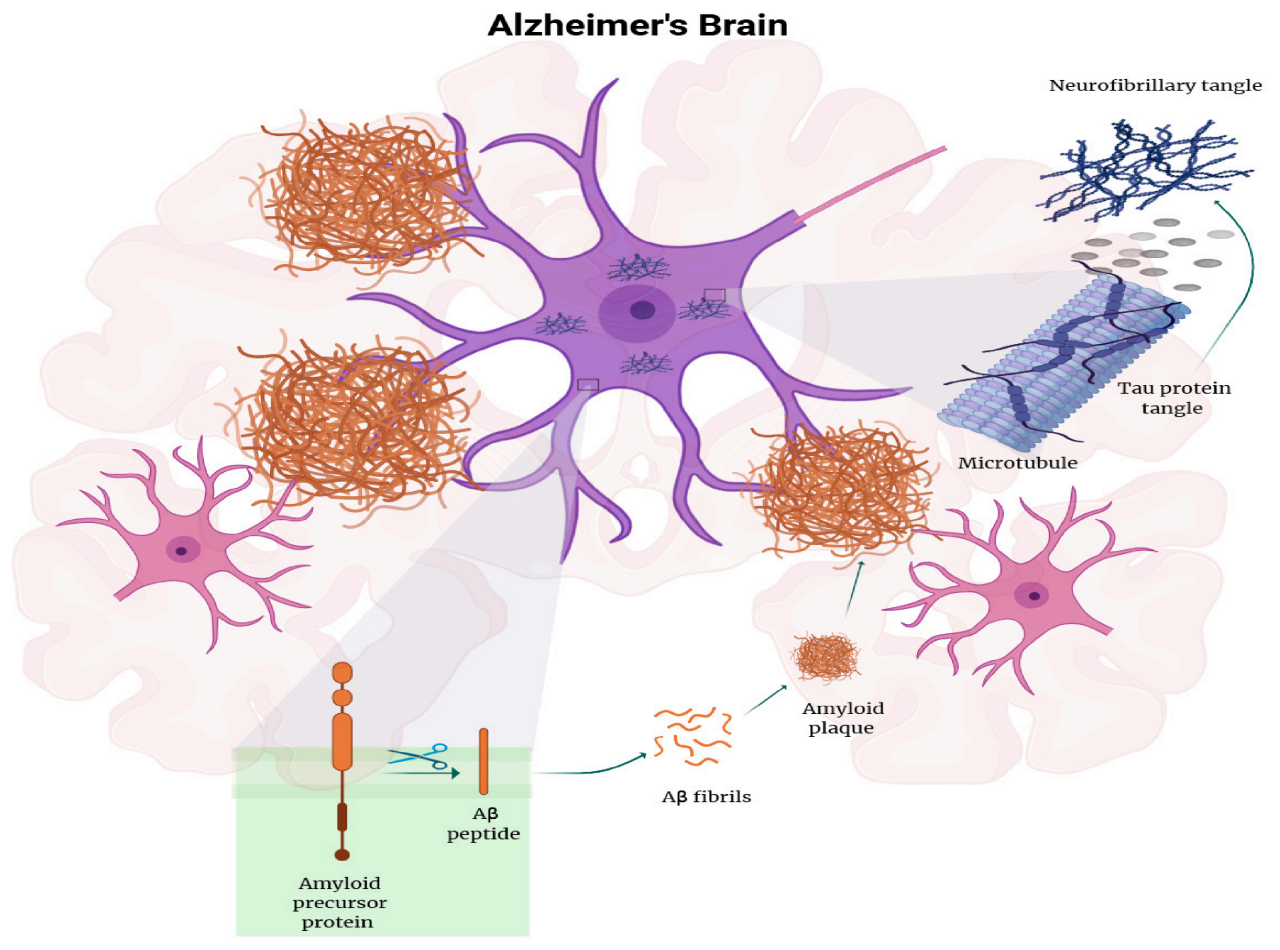
Pathophysiology of Alzheimer’s disease. AD is characterized by the accumulation of abnormal protein aggregates in the brain, primarily in the hippocampus and cortex, which are associated with synaptic dysfunction, neuronal loss, and, consequently, alterations in neuronal circuits. Amyloid plaques are generated by sequential cleavage of the amyloid precursor protein, ultimately releasing toxic Aβ peptides that aggregate extracellularly, assembling senile plaques. On the other hand, hyperphosphorylated Tau protein leads to the formation of insoluble intracellular aggregates, following the formation of structures denominated Neurofibrillary tangles (NFT).

AD is the most common form of dementia in elderly people worldwide. It is estimated that approximately 55 million people have AD, and this number is projected to rise to 130 million by 2050 [26]. Clinically, it is characterized by progressive mental, behavioral, and cognitive declines [27,28] often accompanied by aphasia and agnosia [29]. The major histopathological hallmarks of AD brains include extracellular deposition of amyloid β (Aβ) peptide and intraneuronal accumulation of hyperphosphorylated Tau protein, which produces the formation of senile plaques and neurofibrillary tangles (NFTs), respectively. These protein aggregates cause synaptic dysfunction and neuronal loss, primarily in the hippocampus and cortex, affecting cholinergic circuits and leading to progressive deterioration of memory and cognition [30]. Mechanistically, the amyloid precursor protein is cleaved by two pathways: the non-amyloidogenic pathway and the amyloidogenic pathway [14,31,32]. Under physiological conditions, amyloid precursor protein (APP) is primarily processed by the non-amyloidogenic pathway. Briefly, the transmembrane protein APP is cleaved by α-secretase, generating the C83 fragment and the larger soluble APPα (sAPPα) fragment. Subsequently, it produces the P3 peptide and the APP intracellular domain (AICD) via C83 cleavage by γ-secretase [29,31]. On the other hand, in the amyloidogenic pathway, APP is cleaved by β-site APP-cleaving enzyme 1 (β-secretase, BACE1), forming the soluble APPβ (sAPPβ) fragment and the 99-amino-acid C-terminal fragment (C99) [29,31]. Afterward, γ-secretase cleaves C99 to produce the short hydrophobic peptide Aβ of 40 or 42 amino acids, which assembles and accumulates into insoluble oligomers, protofibrils, fibrils, and in senile plaques observed in AD [14,29] (Figure 2). 

Alzheimer’s therapy has two categories: pharmacological and non-pharmacological. Non-pharmacological treatment includes personal activities and exercise. Pharmacological therapy varies by country and is limited to a few drugs, with supporting evidence available for the management of the disease [33]. The most commonly used combination is acetylcholinesterase inhibitors and N-methyl-D-aspartate (NMDA) receptor antagonists, such as memantine, which has only a minimal impact on disease and has shown symptomatic efficacy without changes in β-amyloid or Tau pathology [33,34,35]. In this scenario, anti-β amyloid monoclonal antibodies are now available as an innovative therapy. Clinical trials demonstrated approximately a 30% slowing of disease progression after 18 months of treatment [36]. However, side effects such as brain edema and hemorrhage were reported; thus, constant MRI monitoring is required [37]. In summary, therapeutic approaches are limited by the disease’s etiological and clinical heterogeneity. Numerous modern pharmacological studies have highlighted the protective effects of natural products and their bioactive compounds, which can target multiple pathways of neurodegenerative diseases [31].

### 2.2. Alzheimer’s Disease Study Models

The generation of models that reproduce features of AD is very important for improving the understanding of this disease and developing new therapies [30]. An in vitro model used for AD studies involves incubating neuronal or microglial cultures with Aβ_1–42_ peptides or oligomers [38,39]. Another alternative is to transfect cultures with plasmids expressing Aβ [19]. Additionally, cell-induced toxicity with aluminum maltolate has been considered as a new option that mimics AD neuropathology [40]. These models reproduce the distinctive characteristics of the disease, such as oxidative stress, inflammation, apoptosis, and microglial activation, and allow the evaluation of the capacity of various agents to inhibit neuronal damage in AD [19,30,40]. In vivo studies are commonly performed in transgenic mice harboring genetic mutations associated with familial AD [41]. The APP/PS1 mouse is a transgenic mouse that expresses a chimeric mouse/human APP with Swedish mutations (K595N/M596L) and the human presenilin 1 with exon 9 deletion (PS1-dE9) [42]. The J20 mouse model overexpresses human APP with the Swedish (K670N/M671L) and Indiana (V717F) mutations [43]. The triple-transgenic AD mouse model (3xTg-AD) develops amyloid and Tau pathology in a temporal sequence. This is a transgenic mouse model that expresses three mutated human genes related to Alzheimer’s disease: APP (K670N/M671L), PS1 (M146V), and Tau (P301L) [41].

On the other hand, the intra-cerebroventricular injection of streptozotocin (ICV-STZ), a glucosamine derivative of nitrosourea used to induce diabetes in rats, has provided a relevant animal model for sporadic AD. These models reproduce AD-like neuropathological features, like accelerated Aβ aggregation, Tau hyperphosphorylation, and impaired brain energy metabolism [44,45,46]. The main contribution of in vivo models is that they allow the study of the effects of different pharmaceutical approaches on memory and cognition [30,47]. A highly valuable natural animal model of AD is the Chilean endemic rodent *Octodon degus*, recognized as the first wild-type rodent model for studying AD pathology [48]. Interestingly, this rodent shares a high sequence homology with human Aβ, differing only in a single amino acid substitution [48]. The rodent *Octodon degus* spontaneously develops AD features with age, including Aβ aggregates, hyperphosphorylation, and intracellular Tau accumulations, and reduced cognitive function [49,50].

### 2.3. Andrographolide Improves Cognition and Memory

To study the effects of ANDRO on memory and cognition, assays related to hippocampal roles were performed in animal models of AD. Aged degus (56 months old) treated with ANDRO improved their performance of novel location recognition (NLR) and novel object recognition (NOR) test than the age-matched control, achieving a Recognition Index similar to young controls (12 months old) [28]. Furthermore, aged degus treated with ANDRO show a shorter time to locate the escape hole in the Barnes Maze test than age-matched controls [28]. ANDRO-treated APP/PS-1 mice have significantly lower escape latency in the Morris water maze (MWM) test than transgenic control, and the values are similar to wild-type mice [30]. Moreover, APP/PS-1 transgenic mice treated with ANDRO, when subjected to the object location memory (OLM) task, spent more time exploring the displaced object than the non-displaced one, whereas APP/PS-1 mice alone explored both objects, indicating no preference for the displaced object [51]. Similarly, short-term spatial memory was assessed in STZ-induced AD-like rats treated with ANDRO using a Y-maze. The results show that ANDRO mitigates the short-term spatial memory impairment [52]. Taken together, these data suggest that ANDRO improves spatial memory and learning performance in AD animal models. 

### 2.4. Decrease in Oxidative Stress and Neuroinflammation

There is growing evidence indicating that oxidative stress and inflammation are linked to several degenerative diseases. The presence of extensive oxidative stress is a characteristic of AD brains, and the imbalance between reactive oxygen species (ROS) and the antioxidant defense system may constitute a plausible pathogenic mechanism for AD [53,54]. It has been determined that ANDRO could scavenge ROS and protect neuronal cells from oxidative damage [55]. Xu et al. showed that ANDRO treatment inhibited ROS production in a microglial cell line, suggesting that this effect may represent a molecular mechanism underlying its neuroprotective action [55]. In animal models, ANDRO treatment led to a marked reduction in the expression of oxidative stress markers, such as N-tyrosine (N-Tyr) and 4-hydroxynonenal (4-HNE), in the hippocampus of aged degus than age-matched controls [53]. Similarly, in STZ-induced AD-like rats, ANDRO reduced malondialdehyde (MDA) levels and increased GSH, SOD, and CAT antioxidant activity [56]. Regarding the neuroinflammation in AD, the BV-2 microglial cell line treated with Aβ_1–42_ shows that ANDRO attenuates the secretion of proinflammatory cytokines like IL-1β, IL-6, and TNF-α [19,57] and downregulates the protein levels of inducible nitric oxide synthase (iNOS) and cyclooxygenase-2 (COX-2) [39]. Furthermore, ANDRO could inhibit activation of the Nuclear Factor κB (NF-κB) transcription signaling pathway, which is essential for the proinflammatory gene expression, such as iNOS, COX-2, TNF-α, and IL-1β [39,57]. NF-κB activation requires phosphorylation of the inhibitor of κB (IκB) via the IκB kinase (IKK). Yang et al. determined that nuclear translocation of NF-κB was inhibited due to the decrease in IκB phosphorylation levels by ANDRO [39]. Moreover, ANDRO reduces JNK-MAPK phosphorylation levels, thereby modulating the production of inflammatory mediators [39].

Recently, it has been determined that ANDRO has anti-inflammatory activity through the modulation of microRNA expression [58]. Specifically, ANDRO reduces proinflammatory cytokines and NF-κBp65 expression and increases the levels of p62 and Microtubule-associated protein 1A/1B-light chain 3-II (LC3II) proteins by downregulating miR-222 in AD models in vitro and in vivo [58]. This microRNA has been reported to increase in patients and models of AD, and has been described as a key molecule in neurodegenerative processes [59]. Therefore, ANDRO could reverse the upregulation of miR-222 in AD, providing new insights into the mechanism of action of ANDRO as a therapeutic agent [59]. Taken together, ANDRO could exert a neuroprotective effect by reducing oxidative stress and inflammation induced by Aβ toxicity in AD models. 

### 2.5. Promotion of Autophagy and Aβ Clearance

Several studies have suggested that the accumulation of protein aggregates in the AD brain is due to a decline in clearance rates rather than its overproduction [60]. Autophagy is the major intracellular degradation pathway for clearing misfolded proteins, damaged organelles, and recycling building blocks to maintain homeostasis in mammalian cells [61,62]. In post-mortem AD patients’ brains, impairments in the maturation of autophagic vacuoles [63] and downregulation of key autophagy regulators, such as Beclin-1, have been observed [64], indicating a deficit in the autophagic process. Hence, promoting autophagy has now been considered a promising therapeutic strategy for AD. In cell models of AD, ANDRO positively modulates autophagy-related genes and proteins, such as Beclin-1, Microtubule-associated protein 1A/1B-light chain 3 (LC3), Nrf2, and p62, inducing Aβ clearance [38,40]. Furthermore, ANDRO decreases the expression of APP and BACE1, reducing Aβ production [40]. In vitro, ANDRO has been reported to increase α-secretase gene expression and activity while decreasing β-secretase activity, thereby raising the sAPPα/βAPP ratio [65].

### 2.6. Decrease in Tau Phosphorylation

NFTs are formed by a hyperphosphorylated microtubule-associated protein known as Tau [66]. The abnormal phosphorylation levels reduce Tau’s ability to bind to microtubules and induce self-assembly into insoluble tangles/filaments [67]. It has been shown in vitro and in vivo that treatment with ANDRO significantly reduces phosphorylation related to pathological aggregation of Tau, such as Ser396 [30,38,40], Thr231 [28], Ser404, Ser202, and Thr205 [30]. Altogether, the evidence indicates that ANDRO could decrease protein aggregation in AD by modulating the expression and activity of proteins involved in Aβ aggregate formation, reducing Tau hyperphosphorylation, and increasing the clearance of insoluble protein aggregates via autophagy.

### 2.7. Hippocampal Neurogenesis

It has been reported that ANDRO promotes hippocampal neurogenesis in APP/PS-1 mice, assessed by the increase in proliferation markers Ki67 and Bromodeoxyuridine (BrdU) in the dentate gyrus. Nevertheless, co-treatment with the anti-mitotic drug Temozolomide does not worsen performance on the object location memory (OLM) task [51], suggesting that other ANDRO effects might underline cognitive improvement.

### 2.8. Wnt/β-Catenin Signaling Modulation

Canonical Wnt/β-Catenin signaling is an important pathway that regulates embryonic development, tissue maturation, and cell homeostasis, among other biological processes [68]. Briefly, in the canonical pathway, when signaling is activated, the Wnt ligand binds to the Frizzled transmembrane receptor and the coreceptor low-density lipoprotein receptor-related protein 5/6 (LRP5/6), which, in turn, inactivates the destruction complex of β-Catenin. This complex comprises adenomatosis polyposis coli (APC), axis inhibition protein (Axin), and two kinases, casein kinase 1α (CK1α) and glycogen synthase kinase-3β (GSK-3β), which phosphorylate the transcriptional cofactor β-Catenin. This allows β-Catenin to accumulate in the cytoplasm and translocate to the nucleus, where it binds to -TCF/LEF transcription factors and activates gene transcription. On the other hand, when Wnt/β-Catenin signaling is inactivated, GSK-3β phosphorylates β-Catenin and induces its degradation by the proteasome, preventing gene transcription [14,46,68,69]. 

In the mature mammalian CNS, this pathway participates in adult neurogenesis and has an important role in modulating synaptic structure and function [69]. Therefore, Wnt/β-Catenin signaling maintains and protects neuronal connections throughout the entire life [69]. In AD, it has been reported that Wnt/β-Catenin signaling activity is decreased in animal models of the disease, accelerating the onset of AD neuropathological characteristics [46]. Specifically, the reduction in Wnt/β-Catenin signaling plays a pivotal role in promoting Tau phosphorylation, Aβ aggregation, and cognitive impairment [28,46]. It has been described that ANDRO treatment reestablishes protein levels of key components of canonical Wnt signaling, such as β-Catenin. Moreover, ANDRO promotes activation of Wnt/β-Catenin signaling and its neuroprotective effects by increasing Ser9 inhibitory phosphorylation of GSK-3β in the hippocampus of old degus and APP/PS-1 mice [30,70].

### 2.9. Reestablishment of Postsynaptic Protein Expression

It has been determined that ANDRO improves hippocampal processes, increasing postsynaptic protein levels. ANDRO treatment in old degus could reverse age-dependent decreases in the expression of the postsynaptic proteins: vesicular glutamate transporter 1 (VGlut1), postsynaptic density protein 95 (PSD-95), and NMDA receptor subunits GluN2A and GluN2B [28,71]. In this line, an increase in postsynaptic proteins, including Pan-Shank, AMPA receptors, and the GluA2 subunit, was observed in APP/PS-1 mice treated with ANDRO [30].

### 2.10. Enhancement of Brain Glucose Metabolism

The reduction in cellular energy metabolism, specifically the dysregulation of glucose metabolism, has been considered a marker of AD progression [72]. Cerebral glucose hypometabolism is a common pathophysiological feature of many neurodegenerative diseases [72]. In AD, this has been postulated as one of the first events of pathology, even when the cognitive impairments have not yet become evident [72]. Modulating glucose metabolism has emerged as an attractive therapeutic target for patients with early-stage AD [43]. Evidence suggests that several ANDRO targets participate in the control of glucose metabolism [73,74]. In transgenic AD mice models, it was found that ANDRO treatment recovers glucose uptake and glycolytic rate by the restoration of Glucose Transporter 3 (GLUT3) expression and increased activity of key enzymes for glycolysis and AMP-activated protein kinase (AMPK) in the hippocampus, similar to wild-type values [43,75]. Consequently, the positive modulation of glucose metabolism is a recovery of ATP production in the brains of transgenic mouse models of AD [43]. Recently, it has been reported that in cortical astrocytes, ANDRO increases glucose uptake and activates glucose-6-phosphate dehydrogenase (G6PDH) enzyme, promoting glucose flux toward the pentose phosphate pathway (PPP) [76]. Additionally, the results showed an increase in the NADH/NADP^+^ ratio and GSH levels, indicating greater antioxidant capacity to maintain redox balance. This effect of ANDRO differs from that observed in neurons, where it increases glycolysis, likely to generate a microenvironment better prepared to respond to oxidative and inflammatory stress [76]. Nevertheless, it is necessary to study this metabolic reprogramming in astrocytes using AD models. Table 1 shows a summary of the possible effects of ANDRO in AD models.

## 3. Protective Actions of ANDRO in Parkinson’s Disease

### 3.1. Pathophysiology of Parkinson’s Disease

**Figure 3:**
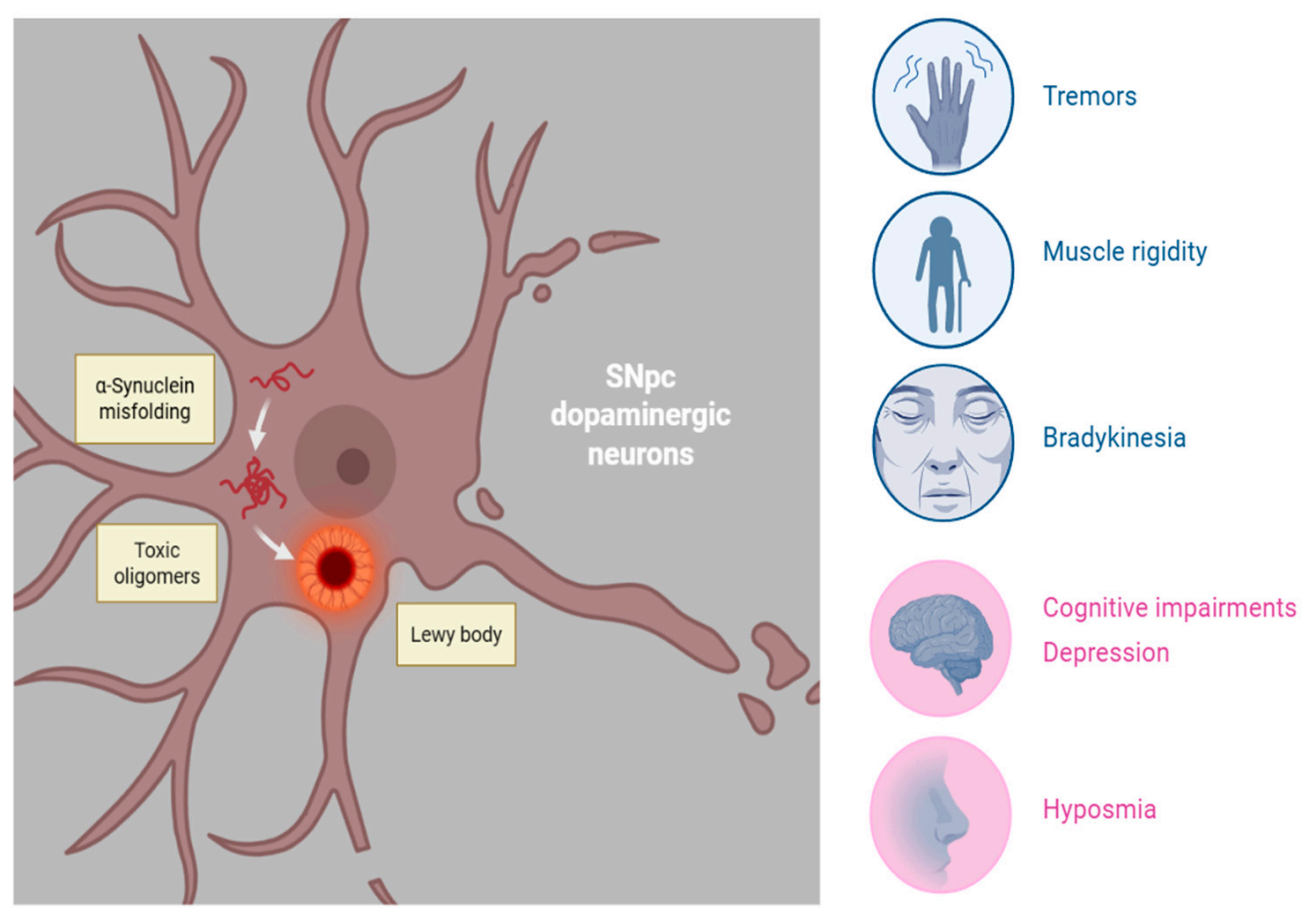
Pathological features in Parkinson’s disease. The hallmark of PD is the intracellular accumulation of misfolded α-syn proteins, which form toxic oligomers that aggregate into Lewy bodies in dopaminergic neurons in the midbrain’s substancia nigra pars compacta (SNpc). These structures drive a progressive loss of dopaminergic neurons, disrupting motor circuits and causing the classic clinical features of PD. The white arrows show the progression of α-syn aggregate formation until the assembly of Lewy bodies. On the right, motor (blue) and non-motor (pink) symptoms are shown.

PD is a chronic and progressive neurodegenerative disease. Currently, PD is the second most prevalent neurodegenerative disorder after AD, affecting 1–3% of the population older than 60 years [77,78]. About 90% of PD cases are idiopathic (sporadic), whereas familial PD accounts for only 10% and is usually linked to genetic mutations [79,80]. The most pronounced neuropathological features in patients are the progressive loss of dopaminergic neurons in the substantia nigra pars compacta (SNpc) and inclusions of the synaptic protein α-synuclein (α-syn), in the cell bodies and processes of surviving neurons, known as Lewy bodies and Lewy neurites, respectively [81]. This leads to a reduction in dopamine levels in the striatum, which causes the characteristic motor symptoms of the disease, such as resting tremor, bradykinesia, and muscle rigidity. In addition, patients may present non-motor symptoms such as cognitive impairments, depression, sleep dysfunction, and hyposmia [79] (Figure 3). 

The traditional strategy to relieve motor symptoms consists of augmenting dopamine levels by administering exogenous dopamine (L-DOPA), monoamine oxidase B inhibitors, and/or dopamine receptor agonists [82,83]. Currently, deep-brain stimulation of the subthalamic nucleus is performed in patients who do not achieve adequate symptom control with traditional pharmacological treatment, resulting in sustained improvements in motor function lasting up to 10 years after surgery [84,85]. On the other hand, selective serotonin reuptake inhibitors and cholinesterase inhibitors are used for non-motor symptoms [86]. 

Several mechanisms have been described to explain neuronal loss in PD; the main ones are neuroinflammation, mitochondrial dysfunction, and impaired protein clearance mechanisms [87]. These pathogenic mechanisms are potential therapeutic targets, and their intervention with low-molecular-weight natural compounds may be a promising approach to prevent disease progression and improve patients’ quality of life. 

### 3.2. Parkinson’s Disease Study Models

Sporadic PD models induced by neurotoxins or pesticides/herbicides are useful tools for elucidating the pathogenetic mechanisms of neurodegeneration in PD [88]. The most used neurotoxins are 6-hydroxydopamine (6-OHDA), 1-methyl-4-phenylpyridinium (MPP^+^), and 1-methyl-4-phenyl-1,2,3,6-tetrahydropyridine (MPTP). These neurotoxins exert deleterious effects on dopaminergic neurons of the SNpc and striatum, mimicking various pathological hallmarks and motor aspects of the disease [89,90]. Importantly, it has been suggested that, related to sporadic PD, environmental factors may lead to α-syn [91,92]. Agrochemicals, such as Rotenone and Paraquat, can drive α-syn overexpression and aggregation in vitro and in vivo [93,94]. In vivo experiments showed that ANDRO administration attenuates the loss of dopaminergic neurons and ameliorates behavioral deficits in MPTP-challenged mice, including tremors, motor coordination, spontaneous movement, and neuromuscular tone [95,96]. The cellular and molecular mechanisms underlying this improvement induced by ANDRO are detailed below.

### 3.3. Regulation of Mitochondrial Dynamics in PD

Disruption of the balance between mitochondrial fission and fusion may lead to reduced mitochondrial motility, decreased energy production, and increased oxidative stress, thereby negatively affecting neuronal function and resulting in cell death [97]. Excessive mitochondrial fission has been implicated in the progressive deterioration of dopaminergic neurons in PD models. Therefore, mitochondrial dynamics-related proteins could be potential targets for PD treatment [32,98,99]. ANDRO treatment in the rotenone-induced PD model has been shown to prevent excessive mitochondrial fission and preserve mitochondrial homeostasis. Mechanistically, ANDRO directly binds to dynamin-related protein 1 (DRP1), inhibiting its GTPase activity and blocking the DRP1 oligomerization. Consequently, high levels of mitochondrial fission are inhibited [96].

### 3.4. Promotion of Mitophagy and Reduction in Inflammation

Inflammasomes are intracellular pro-inflammatory pattern-recognition receptors that can initiate and propagate inflammation [100]. These cellular complexes are well characterized in the innate immune system. It has been described that microglial NLR family pyrin domain-containing 3 (NLRP3) may be a sustained source of neuroinflammation that could drive progressive dopaminergic degeneration [101]. Extensive inflammasome activation and microglial NLRP3 expression have been observed in the SNpc of PD patients and animal models [102,103]. Several pathophysiological factors present in PD, such as mitochondrial dysfunction, impaired mitophagy, and oxidative stress, play a crucial role in activating the NLRP3 inflammasome complex [104,105]. A study in a murine MPTP-induced PD model demonstrates that ANDRO treatment promotes E3 ubiquitin-protein ligase parkin (Parkin) dependent mitophagy, thereby removing defective mitochondria, suppressing NLRP3 inflammasome activation in microglia, reducing inflammation and dopaminergic neuronal loss, and improving behavioral parameters [95]. Similarly, it has been determined that ANDRO enhances mitophagy and autophagy induction, facilitating the clearance of α-syn and damaged mitochondria, in the MPP^+^ SH-SY5Y model. The results showed that ANDRO increases the levels of key autophagy-related proteins Beclin-1, LC3B/A, and Lysosomal-associated membrane protein 1 (LAMP1), which are involved in autophagosome formation. Regarding mitophagy, ANDRO upregulates the expression of PTEN-induced kinase 1 (PINK1) and Parkin, indicating that mitochondria are destined for mitophagy. To determine mitophagy induction, the degree of colocalization between the autophagosome and mitochondrial marker was analyzed using Microtubule-associated protein 1A/1B-light chain 3 (LC3), and Mito Tracker Red, respectively, indicating that ANDRO increases this process [89]. Interestingly, in this PD model, ANDRO promotes the expression of the enzyme tyrosine hydroxylase, which induces dopamine synthesis and activates the antioxidant Nrf2 pathway to provide neuroprotection [89]. 

### 3.5. Modulation of Protein Quality Control Machinery

Cellular proteostasis is essential for maintaining a stable environment for neuronal activity and is ensured by mechanisms including regulation of protein translation, chaperone-assisted protein folding, and protein degradation pathways [106]. It is suggested that a direct link exists between impaired proteostasis and PD etiology. Impaired proteostasis contributes to α-syn aggregate formation in SNpc neurons, which promotes disease progression [107,108]. In vivo studies show that ANDRO administration mitigates MPTP neuronal toxicity by inducing heat shock transcription factor 1 (HSF1) and nuclear Nrf2 expression [109]. Specifically, HSF1 is a master regulator of the heat shock response (HSR) and mediates proteostasis by upregulating chaperone expression, such as HSP70 [110]. The positive regulation of the protein quality control machinery by ANDRO is reflected in increased HSP70 expression, decreased α-syn levels, and reduced neuronal loss observed in the MPTP-treated mouse brain [109].

### 3.6. Potential Therapeutic Target of ANDRO in PD: GSK-3β

GSK-3β is a conserved threonine/serine kinase originally identified as a regulator of glycogen synthase. Currently, GSK-3β is known as a pleiotropic enzyme, as it phosphorylates and regulates more than 50 substrates, acting as a central point in several signaling pathways, thus modulating numerous biological functions [111]. Preclinical and clinical studies determined that dysregulation of GSK-3β is involved in neurodegenerative diseases [80]. In PD, the activity of this enzyme has been found to be elevated in the striatum of postmortem brains [112,113]. Furthermore, GSK-3β phosphorylates Tau protein at residues Ser369/404 and α-syn at Ser129 [112,114]. High levels of these phosphorylations are observed in the respective protein aggregates [115,116], supporting that GSK-3β activation plays a pivotal role in the pathogenesis of PD. Studies in PD models revealed that inhibition of GSK-3β activity prevented dopaminergic neuronal injury, restored striatal dopamine depletion, and ameliorated behavioral impairments caused by MPTP toxicity [117]. In human neuroblastoma SH-SY5Y cells stably expressing α-syn, the inhibition of GSK-3β prevented Tau phosphorylation and reduced α-syn protein levels [118]. In vitro and in silico studies identified ANDRO as a novel inhibitor of GSK-3β through a mechanism that does not involve competition for ATP but rather competition at the substrate-binding site. Furthermore, ANDRO could increase phosphorylation at Ser9, which inhibits its activity [23]. Most known GSK-3β inhibitor compounds compete for the ATP-binding site, whereas ANDRO is a competitive inhibitor at the substrate-binding site, which could offer several advantages. In terms of selectivity, these types of kinase inhibitors are more specific and, therefore, have lower IC50 values, which are necessary to avoid toxicity [119]. In vitro assays demonstrate that substrate-competitive inhibitors perform moderate inhibition. Nevertheless, this may be beneficial, since GSK-3β is a ubiquitous and constitutively active enzyme essential for life [113], and it has been suggested that mild inhibition of the kinase (25–50%) is desirable for treating conditions associated with elevated GSK-3β activity [23,119]. Table 2 shows the molecular mechanisms of ANDRO in PD models.

## 4. ANDRO Derivatives

Currently, various compounds derived from ANDRO have been synthesized by modifying their molecular structures to enhance pharmacological properties, improve bioavailability, and reduce toxicity risk [120,121]. ANDRO derivatives that affect the CNS mainly exhibit modifications to hydroxyl groups [120]. Among the compounds tested in AD models, 19-O-Succinate Dehydrated Andrographolide, known as ANDRO-III, has been shown to enhance cognitive performance, attenuate neuroinflammation, and reduce neuronal damage by down-regulating GSK-3β/NF-κB signaling pathway in 3xTg-AD mice. These neuroprotective effects are greater than those observed with ANDRO [122], and nephrotoxicity is remarkably lower than ANDRO [121].

It has been demonstrated that the compound 3,14,19-Triacetylandrographolide (ADA) prevents cognitive decline and enhances synaptic plasticity in the *Apoe4* sporadic AD mouse model. Furthermore, it could reduce mitophagy dysfunction via upregulation of the SIRT3-FOXO3a pathway and subsequently inhibit NLRP3 inflammasome activation [123]. In the 3xTg-AD mouse brain, ADA treatment increases synaptic protein expression and promotes autophagy by inhibiting the AKT/mTOR pathway [124]. Another ANDRO derivative, andrographolide sulfonate (ANDRO-S), was administered to the APP/PS1 AD mouse prior to the onset of Aβ plaque formation, resulting in decreased neuronal damage and mitochondrial swelling without reducing Aβ deposition in the hippocampus [121,125]. 

In PD models, Andrographolide-lipoic acid (AL-19) reversed MPTP-induced loss of tyrosine hydroxylase-positive neurons, promoting antioxidant activity and decreasing phosphorylated NF-κB [126].

In summary, the use of ANDRO derivatives in AD and PD is an important approach due to their more potent neuroprotective effects than ANDRO treatment [120,121]. Furthermore, certain modifications to the molecule significantly reduce the risk of nephrotoxicity, minimizing potential side effects in patients [121].

## 5. ANDRO Toxicity

Although ANDRO has demonstrated multiple pharmacological activities, mild to moderate adverse effects have been reported in patients following administration of ANDRO and its derivatives, and some of these effects may resolve after discontinuation of the drug. Moreover, an exceptionally limited number of patients experienced serious or life-threatening adverse effects [127,128].

Regarding toxicity at the CNS level, some patients have reported dizziness, headache, and convulsions [127]. Long-term oral administration of ANDRO to Human Immunodeficiency Virus (HIV) positive patients reported adverse events such as decreased short-term memory, dizziness, headache, and fatigue. They also exhibited systemic effects, including pruritus/rash and loose stools/diarrhea, among others [129]. In mice, high doses of ANDRO (5 mg/kg) attenuate middle cerebral artery occlusion/reperfusion-induced brain injury by inducing apoptosis in cerebral endothelial cells and thereby disrupting BBB integrity [130]. However, previous studies have shown that lower doses of ANDRO (0.1 mg/kg) protected against brain injury [131,132].

At systemic levels, in vivo studies showed that repeated administration of a high dose of ANDRO (100 mg/kg) induces damage in the lung, liver, uterus, and kidney, characterized by pulmonary alveolar disruption, renal tubular edema, and elevated serum liver enzymes. Nevertheless, this effect is not observed after administering a single dose [133]. Interestingly, a long-term Chinese patient analysis reported that a small population of patients developed acute kidney injury after one to six doses of intravenous infusion of ANDRO (100–750 mg). The patients exhibit symptoms including flank pain, decreased urine volume, and nausea or vomiting [134]. In vivo studies showed that the mechanisms underlying this harmful effect include induction of senescence in the kidneys and tubular epithelial cells through inhibition of the SIRT3/p53 signaling pathway, resulting in upregulation of downstream target genes involved in inflammation and fibrosis [135].

Regarding reproductive toxicity, ANDRO promotes reproductive dysfunction in animal models [136]. In male rodents, ANDRO causes testicular damage by triggering ferroptosis in Sertoli cells, leading to disruption of the blood-testis barrier and spermatogenic impairment [137]. Additionally, ANDRO induces impaired fertility in female rodents by disrupting oocyte meiotic maturation and blocking cytoskeletal reorganization [138]. In vitro studies in human embryonic stem cells (hESCs) confirmed that ANDRO exhibited notable reproductive toxicity, inducing apoptosis via an oxidative stress response [139].

In conclusion, long-term accumulation, rapid administration, and high doses of ANDRO can trigger adverse events at the CNS and/or systemic levels [140]. Hence, an individual patient evaluation of the clinical features, route of administration, and dose is required. Further studies are needed to ensure their safety in clinical use.

## 6. Conclusions

**Figure 4:**
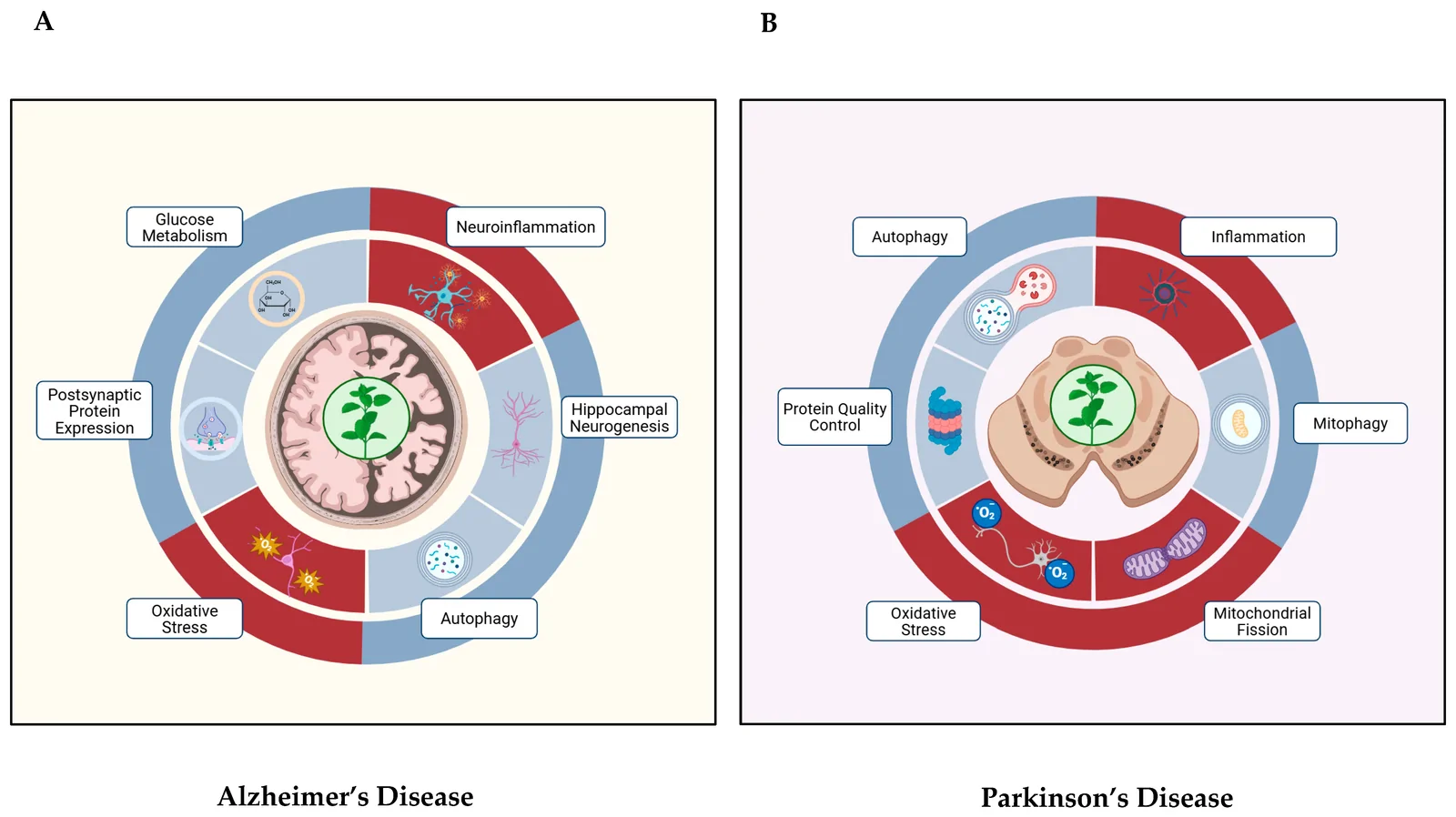
Cellular effects of ANDRO in the CNS in AD and PD. The figure shows the biological effects in which ANDRO is involved in (**A**) AD and (**B**) PD models. Blue boxes indicate the promoting effects of ANDRO, and the red boxes indicate the reducing effects of ANDRO.

The search for natural compounds targeting multiple mechanisms of action, including preventing cell death and restoring function in damaged neurons, could be promising for the prevention and treatment of neurodegenerative diseases. In this review, we summarize the significant neuroprotective potential of ANDRO, a diterpenoid lactone from *A. paniculata*, which has been demonstrated to have multiple biological targets implicated in the progression of the most common neurodegenerative disorders: AD and PD (Table 1 and Table 2). In AD, ANDRO has been shown to decrease the accumulation of protein aggregates by promoting autophagy and reducing protein phosphorylation associated with pathogenicity. In addition, it promotes the recovery of synaptic functions and improves brain energy metabolism, reducing neuroinflammation and oxidative stress. The restoration of hippocampal neuronal function induced by ANDRO is reflected in improved cognition and memory in AD animal models (Figure 4A).

On the other hand, in PD model studies, ANDRO attenuates the loss of dopaminergic neurons by upregulating proteostasis, inhibiting excessive mitochondrial fission, promoting mitophagy, and reducing neuroinflammation (Figure 4B). It is suggested that the decrease in pathological hallmarks induced by ANDRO underlies the behavioral and motor improvements observed in PD models. Interestingly, ANDRO has been reported to be a competitive inhibitor of GSK-3β, a central component of several signaling pathways implicated in PD pathogenesis. Hence, ANDRO’s potential to ameliorate various features of PD and AD in in vitro and in vivo models supports its use as a therapeutic agent. Moreover, ANDRO’s capacity to bind multiple targets is a valuable property for treating diseases with multifactorial etiologies. Nevertheless, the promiscuity of ANDRO needs to be considered to prevent adverse effects. Finally, further studies are needed to verify the neuroprotection observed in preclinical trials and to evaluate doses and possible routes of administration. 

The literature search for this review was conducted in PubMed and Google Scholar. First, a general examination was performed using the keywords “andrographolide” or “ANDRO”, combined with “neurodegenerative diseases”, “Alzheimer’s disease”, “Parkinson’s disease”, and “neuroprotection”. Subsequently, additional keyword combinations targeted specific aspects of ANDRO’s biological activity, in the context of neurodegenerative diseases, including “cognition”, “neuroinflammation”, “oxidative stress”, “autophagy”, “mitophagy”, “mitochondrial dysfunction”, “glucose metabolism”, or “Wnt signaling”. The final dataset comprised 140 sources, mainly original research articles and review studies from the last three decades (1992–2026).

## Figures and Tables

**Table 1:** Neuroprotective effects of ANDRO in AD study models. The table describes the studies that determined the effect of ANDRO on AD. It indicates the study reference, the AD model, the type of ANDRO treatment, and the neuroprotective effect of ANDRO. I.P: Intraperitoneal.

Reference	AD Model	Treatment	Neuroprotective Effect
Arredondo et al., 2021 [51]	APP/PS1 mice (8 months old)	I.P. injections of 2.0 mg/kg ANDRO, 3 times per week for 4 weeks.	Promotion of neurogenesis.
Cisternas et al., 2019 [43]	J20 mice (3 months old)	I.P. injections of 2.0 mg/kg ANDRO, 3 times per week for 16 weeks.	Increases glycolysis enzyme activity.
Gherardelli et al., 2022 [75]	APP/PS1 mice Female and male. (4, 8 months and 11 months old)	I.P. injections of 2.0 mg/kg ANDRO, 3 times per week for 4 months.	Restores glycolytic and pentose phosphate pathway fluxes. Increases expression of canonical Wnt/β-Catenin signaling target genes.
Gu et al., 2018 [38]	PC-12 cells Treatment with 10 μM Aβ_1–42_ for 24 h	Pre-treatment with 20 μM ANDRO for 1 h.	Modulation of the Nrf2-mediated p62 autophagy signaling pathway and decrease in Tau hyperphosphorylation.
Inestrosa et al., 2020 [70]	*Octodon degus* (Adult: 24–48 months, Aged: 60–72 months)	I.P. injections of 2.0 or 4.0 mg/kg ANDRO, 3 times per week for 3 months.	Modulation of Canonical Wnt/β-Catenin signaling.
Lu et al., 2021 [40]	PC-12 cells Treatment with 700 μM aluminum maltolate	Co-treatment with 5 or 10 μM ANDRO for 24 h.	Modulation of Nrf2-mediated p62 autophagy signaling pathway, decreases in Tau hyperphosphorylation, and downregulation of BACE1.
Oliva et al., 2023 [71]	Female degus (Adult: 36 months old, Aged: 72 months old)	I.P. injections of 2.0 mg/kg ANDRO, 3 times per week for over 12 months.	Upregulation of postsynaptic protein expression.
Patel et al., 2021 [56]	Wistar rats Treatment with ICV STZ injections (3 mg/kg)	Oral administration of 15, 30, or 60 mg/kg ANDRO for 14 days.	Reduction in TNF-α, IL-1β, and IL- 6 levels, AChE activity, Aβ levels, and Tau hyperphosphorylation.
Rivera et al., 2016 [28]	Female degus (Young: 12 months old, Aged: 56 months old)	I.P. injections of 2.0 or 4.0 mg/kg ANDRO, 3 times per week for 4 weeks.	Decrease in Tau hyperphosphorylation and upregulation of postsynaptic protein expression.
Serrano et al., 2014 [30]	APP/PS1 mice (8 months old)	I.P. injections of 2.0 mg/kg ANDRO, 3 times per week for 4 weeks.	Modulation of Canonical Wnt/β-Catenin signaling and upregulation of post-synaptic protein expression.
Wang et al., 2025 [58]	PC12 cells Treatment with 4 μg/mL LPS for 24 h BV2 cells Treatment with 1 μM Aβ_1–42_ for 24 h 3xTg-AD Male mice (age not reported)	Co-treatment with 10 μM ANDRO for 24 h. I.P. injections of 2 mg/kg ANDRO, 3 times per week for 3 months.	Downregulation of miR-222. Decrease in NF-κB p65 protein levels. Increase expression levels of autophagy markers p62 and the LC3-II/LC3-I ratio. Reduces IL-1β and IL-6 expression levels and increases SOD content in mouse serum.

**Table 2:** Neuroprotective effects of ANDRO in PD study models. Table 2 describes the studies that determined the effect of ANDRO on PD. It indicates the study reference, the AD model, the type of ANDRO treatment, and the neuroprotective effect of ANDRO.

Reference	AD Model	Treatment	Neuroprotective Effect
Ahmed et al., 2021 [95]	N9 cells Treatment with 100 μM of MPP^+^ for 8 h C57BL/6 mice Male (10–12 weeks old). I.P. MPTP 25 mg/kg/day for 5 days	Pretreatment with ANDRO 10 mM for 8 h. Treatment with 5 mg/kg and 10 mg/kg ANDRO daily for 14 days.	Inhibit NLRP3 inflammasome activation. Upregulation of parkin-dependent mitophagy. Rescues dopaminergic neuron loss.
Dutta et al., 2021 [109]	Swiss albino mice Male (4–6 weeks old) Treated with MPTP 20 mg/kg/day Five applications on alternate days	Treatment with 10 mg/kg/day ANDRO. Ten applications in 20 days.	Activates HSF1, upregulating chaperone expression. Induces mTORC1-dependent stabilization of Nrf2. Increases autophagy. Restores TH levels. Decreases α-syn levels.
Geng et al., 2019 [96]	SH-SY5Y cells Treatment with 30 μM of rotenone for 6 h. C57BL/6 mice Male (10–12 weeks old) I.P. MPTP 25 mg/kg/day for 5 days	Pretreatment with 0.1, 0.3, and 1 μM ANDRO for 6 h. Treatment with 2.5 or 5 mg/kg ANDRO for 12 days.	Decreases excessive mitochondrial fission by inhibiting the GTPase activity of DRP1. Mitigates bradykinesia. Enhances neuronal survival and mitochondrial functions.
Prasertsuksri et al., 2023 [89]	SH-SY5Y cells Treatment with 1.5 mM MPP^+^ for 24 h	Pretreatment with 1.5 μM ANDRO for 24 h.	Increases mitophagy and autophagy. Activates Nrf2 to counteract ROS. Attenuates apoptosis.

## Data Availability

Further inquiries can be directed to the corresponding author.

## Abbreviations

The following abbreviations are used in this manuscript:

3xTg-AD	Triple-transgenic Alzheimer’s disease mouse model
4-HNE	4-Hydroxynonenal
6-OHDA	6-Hydroxydopamine
AD	Alzheimer’s disease
AICD	APP intracellular domain
ANDRO	Andrographolide
APC	Adenomatosis polyposis coli
APP	Amyloid Precursor Protein
Axin	Axis inhibition protein
Aβ	Amyloid-β
BACE1	β-Secretase
BBB	Blood–brain barrier
BrdU	Bromodeoxyuridine
C99	99 amino acid C-terminal fragment
CAT	Catalase
CK1α	Casein kinase 1α
CNS	Central nervous system
COX-2	Cyclooxygenase-2
G6PDH	Glucose-6-phosphate dehydrogenase
GSH	Glutathione
GSK-3β	Glycogen synthase kinase-3β
hESCs	Human embryonic stem cells
HSF1	Heat shock transcription factor 1
HSR	Heat shock response
ICV-STZ	Intracerebroventricular injection of streptozotocin
IκB	Inhibitor of κB
IKK	IκB kinase
iNOS	Inducible nitric oxide synthase
LAMP1	Lysosomal-associated membrane protein 1
LC3	Microtubule-associated protein 1A/1B-light chain 3
LRP5/6	Low-density lipoprotein receptor-related protein 5/6
MDA	Malondialdehyde
MPP+	1-Methyl-4-phenylpyridinium
MPTP	1-Methyl-4-phenyl-1,2,3,6-tetrahydropyridine
MWM	Morris water maze
NFTs	Neurofibrillary tangles
NLR	Novel location recognition
NF-κB	Nuclear Factor κB
NLRP3	NLR family pyrin-domain-containing 3
NMDA	N-methyl-D-aspartate
NOR	Novel object recognition
Nrf2	Nuclear factor erythroid 2-related factor 2
N-Tyr	N-tyrosine
OLM	Object location memory
Parkin	E3 ubiquitin-protein ligase parkin
PD	Parkinson’s disease
PINK1	PTEN-induced kinase 1
PPP	Pentose phosphate pathway
PSD-95	Postsynaptic density protein 95
ROS	Reactive oxygen species
sAPPα	Soluble APPα
sAPPβ	Soluble APPβ
SNpc	Substantia nigra pars compacta
SOD	Superoxide dismutase
VGlut1	Vesicular glutamate transporter 1
α-syn	α-Synuclein
